# Causes of death among street-connected children and youth in Eldoret, Kenya

**DOI:** 10.1186/s12914-018-0160-8

**Published:** 2018-05-15

**Authors:** Lonnie Embleton, David Ayuku, Dominic Makori, Allan Kamanda, Paula Braitstein

**Affiliations:** 1 Institute of Medical Science, University of Toronto, Toronto, Canada; 2 College of Health Sciences, School of Medicine, Department of Behavioral Sciences, Moi University, Eldoret, Kenya; 3 Moi Teaching and Referral Hospital, Eldoret, Kenya; 4 College of Health Sciences, School of Medicine, Department of Medicine, Moi University, Eldoret, Kenya; 5 Dalla Lana School of Public Health, University of Toronto, Toronto, Canada; 6 Regenstrief Institute Inc., Indianapolis, USA; 7 Fairbanks School of Public Health, Indiana University, Indianapolis, USA; 8 Division of Epidemiology, 155 College Street, Toronto, ON M5T 3M Canada

**Keywords:** Street children and youth, Mortality, HIV, Kenya, Homicide, Assault, children’s rights

## Abstract

**Background:**

Street-connected young people carry a disproportionate burden of morbidities, and engage in a variety of practices that may heighten their risk of premature mortality, yet there are currently no reports in the literature on the rates or risk factors for mortality among them, nor on their causes of death. In low- and middle-income countries they are frequently in situations that violate their human rights, likely contributing to their increased burden of morbidities and vulnerability to mortality. We thus sought to describe the number of deaths annually, causes of death, and determine the number of deaths attributable to HIV among street-connected young people aged 0 to 30 years in Eldoret, Kenya.

**Methods:**

Eldoret, Kenya has approximately 1900 street-connected young people. We collected data on deaths occurring from October 2009 to December 2016 from Moi Teaching and Referral Hospital records, Academic Model Providing Access to Healthcare HIV program records, and utilized verbal autopsies when no records were available. Descriptive analyses were conducted stratified by sex and age category, and frequencies and proportions were calculated to provide an overview of the decedents. We used logistic regression to assess the association between underlying cause of death and sex, while controlling for age and location of death.

**Results:**

In total there were 100-recorded deaths, 66 among males and 34 among females; 37% of were among those aged ≤18 years. HIV/AIDS (37%) was the most common underlying cause of death, followed by assault (36%) and accidents (10%) for all decedents. Among males, the majority of deaths were attributable to assault (49%) and HIV/AIDS (26%), while females primarily died due to HIV/AIDS (59%).

**Conclusion:**

Our results demonstrate a high number of deaths due to assault among males and HIV/AIDS among males and females. Our findings demonstrate the need for studies of HIV prevalence and incidence among this population to characterize the burden of HIV, particularly among young women given the higher number of deaths attributed to HIV/AIDS among them. Most deaths were preventable and require the urgent attention of service providers and policymakers to implement programs and services to prevent premature mortality and uphold children’s rights.

## Background

In many low- and middle-income countries, the streets play a central role in the lives of children and youth (street-connected young people) [1]. Street-connected young people spend the majority of their days or days and nights on the streets in public places, generating livelihoods, and living in precarious inadequate conditions with a lack of protection. Young people turn to the streets for survival, as a result of enduring poverty, abuse, and family conflict at home [2]. Once on the streets, these young people are highly marginalized, subject to numerous human rights violations [3–5], and carry a disproportionate burden of morbidities in the areas of substance use [6], infectious diseases, mental health, sexual and reproductive health [7, 8]. They frequently experience violence and abuse on the streets, which is perpetrated by peers, family, other adults, and police [7]. All of these factors may heighten their risk of death, yet there are currently no reports in the literature on the rates or risk factors for mortality among street-connected children and youth in low- and middle-income countries, nor on their causes of death [7].

Recently, the UN Office of the High Commissioner Human Rights (UNOHCR) has drafted a General Comment on children in street situations, recognizing their unique circumstances and the need to ensure the Convention on the Rights of the Child (CRC) is adequately protecting street-connected children and youth. The CRC outlines the basic human rights children are entitled to including: non-discrimination; the right to survival; to reach their full potential; to protection from all forms of abuse, neglect, and exploitation; and to participate fully in matters affecting their social, economic, religious, cultural and political life [9]. The UNOHCR identified five key articles of the CRC in relation to children in street situations for the General Comment including: Article 2.1, States Parties shall respect and ensure the rights set forth in the present Convention to each child within their jurisdiction without discrimination of any kind, irrespective of the child’s or his or her parent’s or legal guardian’s race, colour, sex, language, religion, political or other opinion, national, ethnic or social origin, property, disability, birth or other status; Article 6, States Parties recognize that every child has the inherent right to life and shall ensure to the maximum extent possible the survival and development of the child; Article 15, States Parties recognize the rights of the child to freedom of association and to freedom of peaceful assembly; Article 20, A child temporarily or permanently deprived of his or her family environment, or in whose own best interests cannot be allowed to remain in that environment, shall be entitled to special protection and assistance provided by the State. States Parties shall in accordance with their national laws ensure alternative care for such a child; Article 27, States Parties recognize the right of every child to a standard of living adequate for the child’s physical, mental, spiritual, moral and social development [10]. Street-connected young people in low- and middle-income countries are frequently in situations that violate these basic human rights [3–5, 11, 12], likely contributing to their increased burden of morbidities and vulnerability to mortality, which is undoubtedly avoidable in many circumstances.

In North America, homeless youth sustain significantly higher rates of mortality in comparison to their peers in the general population [13–15]. In Canada, Roy et al. found that a cohort of homeless youth had a mortality rate which was 11 times higher than the observed rate among youth nationally [13]. In a subsequent cohort they found mortality decreased substantially after the introduction of housing and social services [14], in alignment with CRC Article 20 and 27, ensuring children and youth have special protection and an adequate standard of living [9]. Most recently, Auerswald [15], found that homeless youth in San Francisco had a mortality rate 10 times that of the general population. The most common causes of death among homeless youth in these studies were suicide and drug-related [13–15]. In Canada, HIV infection was a significant predictor of mortality for street youth [13]. Homeless young people in North America have documented higher rates of HIV in comparison to the general adolescent [16–20] largely due to injection drug use [21–23]. As street-connected children and youth in low- and middle-income countries primarily use volatile solvents, with little to no injection drug use [6], little attention has been paid to their burden of HIV or mortality, despite often living in HIV endemic settings, engaging in high risk sexual practices [24–27], and having numerous morbidities [7].

It is vital to understand street-connected young people’s causes of death and the burden of HIV among them in low- and middle-income countries if deaths are to be prevented and policy and services designed to assist them in accord with the CRC. We thus sought to describe the number of deaths annually, causes of those deaths, and determine the number of deaths attributable to HIV among street-connected young people aged 0–30 in Eldoret, Kenya from October 2009 to December 2016. We chose to expand our definition of young people from the UN definition for ‘youth’ aged 15 to 24 years [28] to encompass children and youth aged 0 to 30 years given the difficulty confirming age in this population in a resource-constrained setting. We also explored differences in causes of death by sex and age group. This study will provide the first report in the literature concerning mortality among street-connected young people in low- and middle-income settings and illuminate the burden of disease among street-connected young people in Eldoret, Kenya.

## Methods

### Study setting

Uasin Gishu (UG) County is located 375 km northwest of Kenya’s capital, Nairobi. In 2010, UG County had a population of 894,179, of whom 41.5% were aged 14 years or less. Approximately 51.3% of the UG County population lives below the Kenyan poverty line. Eldoret town is the administrative capital of UG County and from the 2009 census, had a population of 289,389 [29]. Eldoret is home to Moi University, Moi Teaching and Referral Hospital (MTRH), and the Academic Model Providing Access to Healthcare (AMPATH) [30]. AMPATH was initiated in 2001 as a partnership between MTRH (Kenya’s second referral hospital. A referral hospital refers to a National hospital that has specialized care.), Moi University School of Medicine, and a consortium of universities from North American led by Indiana University. AMPATH began as an HIV care and treatment program, and with support from PEPFAR currently has over 80,000 HIV infected patients in care across a swathe of western Kenya. The MTRH mortuary serves UG County, and deaths that occur outside of hospital are typically brought to MTRH mortuary.

### Study population

Street-connected young people were defined as individuals aged 0 to ≤30 years who either a) were spending both days and nights on the streets, and had limited-to-no parental/guardian contact or b) were spending a portion or majority of their time on the street and had a parent/guardian/caregiver to whom they returned at night. In 2016, there were 1903 individuals counted who were connected to the streets, including 1151 aged less than or equal to 24 years, and 766 aged less than or equal to 18 years in Eldoret as documented through a point-in-time count; of whom 75% were male and 25% were female [31]. It is believed there are between 1000 and 3000 street-connected people in and around Eldoret at any time and that this number varies with seasonal changes and migration patterns in this highly mobile population.

### Sources of data

A community advocate with an in-depth knowledge of Eldoret’s street-connected young people informally documented deaths and conducted unstandardized verbal autopsies from October 2009 to May 2013 and brought this data to our research team. Deaths involving street-connected young people in Eldoret are brought to the attention of the community advocate by barrack leaders (leaders of groups of street youth) or street-connected young people themselves, as they seek his assistance in navigating the burial process for the deceased individuals. In most cases, for deaths that have occurred outside the hospital, at the barracks or in the streets, the community advocate liaises with the police department to transport the deceased to the hospital mortuary. The community advocate’s documentation was provided to the research team and it supplied the names of deceased street-connected young people from October 2009 – May 2013. Completeness of the data from 2009 to 2013 provided by the community advocate may be limited given the unstandardized documentation, however, given the community advocate’s integral role in liaising with the MTRH mortuary, police, and street-connected young people to facilitate autopsies and burials, it is likely the data is reliable and accurate, yet this documentation may have missed deaths that were not reported to the advocate. We sought to retrospectively ascertain detailed data on the documented deaths by using the names, age, sex, and documented cause of death of the deceased provided by the advocate. We searched MTRH mortuary and hospital records to ascertain cause of death for all in and out of hospital deaths from October 2009 to May 2013.

From May 2013 to December 2016 we prospectively collected mortality data and introduced standardized mortality data collection forms (for infants < 1 year and street-connected young people > 1 year) for the community advocate to use to conduct verbal autopsies and retrieve data from the mortuary and hospital records at MTRH. Data collected from the standardized mortality forms included: date of death, name, age, sex, status on the streets, barracks, location of death (in or out of hospital), autopsy performed (yes versus no), circumstances of death, immediate cause of death, underlying cause of death, cause of death data source, history of previously known medical conditions, and history of injuries and accidents. Cause of death was determined based on an account of the circumstances of death and a cause given by next of kin, friends, or barracks leaders when the decedent did not pass through MTRH mortuary. In cases where the community advocate was liaising with the MTRH mortuary, the circumstance of death, immediate cause of death, and underlying cause of death was documented by the advocate from mortuary records from 2013 onwards when possible.

Retrospective and prospective data were compiled into a database and immediate and underlying causes of death were coded as ascertained from MTRH mortuary certificate for cause of death. When no formal records were located, the cause of death reported in this analysis was based on informal verbal autopsies performed by the community advocate in his documentation. Assault was defined as any intentional physical attack sustained by the decedent and may have been the result of mob justice or homicide. Mob justice was defined as punishment enacted by citizens outside of the legal system, on an individual whom a mob claims perpetrated a crime [32]. Mob justice typically results in a violent attack or assault causing bodily harm on the accused as punishment or retribution for a crime. Accidental deaths were defined as unintentional injuries, and included road traffic accidents, drowning, and other accidents. For all deaths from October 2009 to December 2016, AMPATH records were searched to determine the HIV status of the deceased, if they were in care, and on treatment.

### Ethics

Moi University School of Medicine and MTRH Institutional Research Ethics Committee (IREC) approved this study. The community advocate obtained verbal consent from persons providing verbal autopsy information. IREC approved the use of verbal autopsy data collected, and the extraction of hospital, mortuary and other relevant data to help ascertain cause of death.

### Analysis

Descriptive analyses were conducted and frequencies and proportions were calculated to provide an overview of the decedents. We used logistic regression to assess the association between underlying cause of death and sex, while controlling for age and location of death. The statistical program R was used for analysis.

## Results

**Fig. 1:**
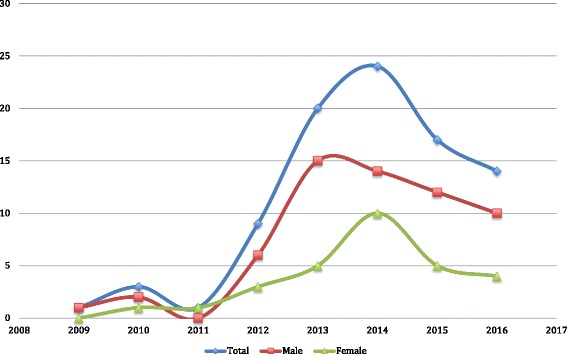
Number of deaths among street-connected young people in Eldoret, Kenya stratified by sex from October 2009 to December 2016

From October 2009 to December 2016, there were 100-recorded deaths among street-connected young people; with 37% of deaths occurring among youth aged ≤18 years (16% among children < 13 years), 66% occurring among males and 34% among females. The median age of decedents was 22 years (IQR: 15–25). Figure 1 demonstrates the number of deaths over time stratified by sex, and shows an increase in recorded deaths with the introduction of standardized mortality data collection from 2013 onwards. Deaths occurred in (38%) and out (48%) of hospital, but more frequently in hospital (56%) for females and out of hospital for males (58%). Less than half (32%) of all decedents had an autopsy. In 70% of cases, immediate and underlying cause of death was established through mortuary and hospital records, with the remaining ascertained through verbal autopsy. Table 1 summarizes the immediate causes of death and Table 2 summarizes the underlying cause of death stratified by sex and age for street-connected young people. Most commonly, HIV/AIDS (37%) was the underlying cause of death, followed by assault (36%) and accidents (10%) for all decedents. Assault was attributed to mob justice in 42% of assault-related deaths that provided details regarding circumstances of death. Among males, the majority of deaths were attributable to assault (49%) and HIV/AIDS (25.8%), while females primarily died due to HIV/AIDS (59%). For decedents aged 18 years or less, assault accounted for 30% of deaths, and the proportion of those that died due to HIV/AIDS was similar between those aged 18 years or less or over 18.

**Table 1:** Immediate cause of death by sex and age among street-connected young people in Eldoret Kenya 2009–2016

Immediate Cause of death	All		Males		Females		≤ 18 years of age		> 18 years of age	
	*N* = 100	%	*n* = 66	%	*n* = 34	%	*n* = 37	%	*n* = 60	%
Blunt Force Trauma	28	28.0%	27	40.9%	1	2.9%	7	18.9%	20	33.3%
Tuberculosis	19	19.0%	8	12.1%	11	32.4%	7	18.9%	12	20.0%
Asphyxia	10	10.0%	9	13.6%	1	2.9%	5	13.5%	5	8.3%
HIV/AIDS	7	7.0%	3	4.5%	4	11.8%	4	10.8%	3	5.0%
Pneumonia	5	5.0%	3	4.5%	2	5.9%	3	8.1%	1	1.7%
Meningitis	4	4.0%	3	4.5%	1	2.9%	1	2.7%	3	5.0%
Infanticide	3	3.0%	0	0.0%	3	8.8%	3	8.1%	0	0.0%
Cardiovascular	3	3.0%	1	1.5%	2	5.9%	0	0.0%	3	5.0%
Diabetes Related	3	3.0%	1	1.5%	2	5.9%	0	0.0%	3	5.0%
Gun shot wound	2	2.0%	2	3.0%	0	0.0%	1	2.7%	1	1.7%
Drug Related	2	2.0%	2	3.0%	0	0.0%	0	0.0%	2	3.3%
Kidney Failure	2	2.0%	1	1.5%	1	2.9%	1	2.7%	1	1.7%
Anaphylaxis	1	1.0%	1	1.5%	0	0.0%	0	0.0%	1	1.7%
Anemia	1	1.0%	0	0.0%	1	2.9%	1	2.7%	0	0.0%
Burns	1	1.0%	1	1.5%	0	0.0%	0	0.0%	1	1.7%
Cerebral Palsy	1	1.0%	0	0.0%	1	2.9%	1	2.7%	0	0.0%
Cervical Cancer	1	1.0%	0	0.0%	1	2.9%	0	0.0%	1	1.7%
Fetal Alcohol Syndrome	1	1.0%	0	0.0%	1	2.9%	1	2.7%	0	0.0%
GI bleeding	1	1.0%	1	1.5%	0	0.0%	0	0.0%	1	1.7%
Liver Cirrhosis	1	1.0%	1	1.5%	0	0.0%	0	0.0%	0	0.0%
Neonatal Sepsis	1	1.0%	0	0.0%	1	2.9%	1	2.7%	0	0.0%
Premature baby	1	1.0%	0	0.0%	1	2.9%	1	2.7%	0	0.0%
Septicaemia	1	1.0%	1	1.5%	0	0.0%	0	0.0%	1	1.7%
Other Traumatic Injury	1	1.0%	1	1.5%	0	0.0%	0	0.0%	1	1.7%

**Table 2:** Underlying cause of death by age and sex among street-connected young people in Eldoret Kenya 2009–2016

Underlying Cause of death	All		Males		Females		≤ 18 years of age		> 18 years of age	
	*N* = 100	%	*n* = 66	%	*n* = 34	%	*n* = 37	%	*n* = 60	%
HIV/AIDS	37	37.0%	17	25.8%	20	58.8%	13	35.1%	23	38.3%
Assault	36	36.0%	32	48.5%	4	11.8%	11	29.7%	24	40.0%
Accidental	10	10.0%	9	13.6%	1	2.9%	4	10.8%	6	10.0%
None Reported	8	8.0%	5	7.6%	3	8.8%	3	8.1%	4	6.7%
Premature baby	2	2.0%	1	1.5%	1	2.9%	2	5.4%	0	0.0%
Diabetes	2	2.0%	0	0.0%	2	5.9%	0	0.0%	2	3.3%
Pneumonia	2	2.0%	1	1.5%	1	2.9%	2	2.7%	0	0.0%
Fetal Alcohol Syndrome	1	1.0%	0	0.0%	1	2.9%	1	2.7%	0	0.0%
Cerebral Palsy	1	1.0%	0	0.0%	1	2.9%	1	0.0%	0	0.0%
Meningitis	1	1.0%	1	1.5%	0	0.0%	0	5.4%	1	1.7%

In total, 37 street-connected young people died due to HIV/AIDS. A total of 22 (59%) had an AMPATH identifying number (meaning that they had at least registered in the AMPATH program), and 16 (43%) had an initial clinical encounter. Of those that had enrolled and had an initial encounter, 7 were in care at their time of death, and 9 were considered lost to follow-up and had not had a clinical encounter within 6 months of their documented date of death. Males were significantly less likely to die due to HIV/AIDS (OR = 0.24 95%CI: 0.10–0.58) in comparison to females, but were significantly more likely to succumb to death due to assault (OR = 7.06, 95% CI: 2.45–25.76). In adjusted analyses controlling for age and location of death these associations remained (HIV/AIDS AOR = 0.18 96%CI: 0.06–0.49, Assault AOR = 5.45, 95%CI: 1.75–20.97).

## Discussion

Our results demonstrate a high number of deaths due to assault among males and HIV/AIDS among males and females. These results suggest that street-connected young people are extremely vulnerable to preventable causes of death. Our findings on mortality among street-connected young people reveal a similar distribution of causes of death to those reported on adolescents in western Kenya, with females succumbing to death due to infectious diseases and males due to injuries [33]. Assault and accidental deaths attributed to almost half of all deaths among street-connected young people, almost exclusively among males and this is significantly higher than reported in community-based adolescents in western Kenya [33] and homeless youth in North America [13, 15]. This high level of homicide is reflective of the reported violence sustained by street-connected young people in Africa [34–37], human rights violations, and lack of policy to protect children and youth in street circumstances [3–5, 12]. Notably, we identified that a large proportion of assault-related deaths among street-connected young people were attributed to mob justice. Mob justice in Kenya is extra-legal punishment enacted by citizens on an individual whom they claim perpetrated a crime in light of a dysfunctional traditional legal system [32]. Due to street-connected young people’s marginalization and discrimination as juvenile delinquents [3, 4, 38], it is likely that in many cases they may be targeted as the perpetrators’ of a crime they did not commit, highlighting the need to uphold street children’s rights to protection from harm and non-discrimination. Moreover, there is extensive documentation in Kenya regarding the maltreatment of street-connected young people by Kenyan authorities resulting in bodily harm and death [4, 39–41]. Local media reported that street-connected children and youth were being forcibly removed by UG county officials and left in neighbouring western counties [39], with a lack of regard for their rights, and provision of protection and care of children as outlined in the Kenya Children’s Act [42], leaving them highly vulnerable. Most recently, international media described the phenomenon of cleaning and clearing the streets of children in UG County and raids that led to the death of several street-connected young people in Eldoret [41].

This study demonstrates that systematic studies of HIV prevalence and incidence among this population are urgently needed to accurately assess the burden of HIV among street-connected young people living in HIV endemic settings. Our preliminary findings should be interpreted with caution, but they suggest a potentially hidden epidemic of HIV among street-connected young people in Eldoret and may indicate a low uptake of and retention in care after testing HIV-positive. Preliminary estimates suggest 4–6% prevalence among street-connected young people in western Kenya [25, 43], exceeding the National prevalence among youth 15–24 (2.1%) [44], and the prevalence found among young people in western Kenya [45]. In this study, the majority of deceased street-connected young people who were HIV-positive did not enroll in care at AMPATH or did not return for care after an initial visit. This highlights the need to develop approaches to strengthen linkage and retention in care for marginalized adolescents who are testing HIV-positive in this region. The limited number of young people enrolling and being retained in care may be reflective of the numerous barriers to adolescent sexual and reproductive healthcare in Kenya that have been documented [46–48]. Children in street circumstances have the right to health and access healthcare (CRC Article 24), and our findings suggest there may be significant barriers to accessing care given the low uptake of registration and retention in care for HIV positive decedents. Given street-connected young people’s socioeconomic marginalization [49], substance use [50, 51], and sexual risk practices [25, 27, 35, 52] in our setting, HIV care and treatment programs will likely need to be specially tailored to respond to the population’s needs.

This study has several limitations. First, the accuracy of the community advocate’s recorded death data from October 2009–May 2013 may be limited, and it is highly probable that deaths occurred that were not recorded. Second, the data was ascertained from a relatively small geographic region of Kenya and therefore may not be representative of street-connected young people throughout the country. Third, cause of death may have been erroneously reported through informal verbal autopsies and we were unable to ascertain data on who provided the cause of death data to the community advocate. We could not assess the accuracy of the information ascertained in the verbal autopsy or if it was collected in a standardized manner prior to 2013. However, due to the relationship the community advocate has with the street-connected young people, it is likely he ascertained relatively accurate data on cause of death due to the trust and respect they place in this individual. It is possible that in some cases the information ascertained may be inaccurate if the person providing data had misinformation regarding the deceased individual. Fourth, individuals who died out of hospital that were not brought to MTRH mortuary had no official record of death. Fifth, In some cases it is likely we missed cases in the morgue master registry as street-connected children and youth are often documented as “unidentified African male/female” and if no one claims the deceased they are disposed of with little documentation remaining concerning their death. It was impossible in some cases to match the documentation in the morgue records to the documentation provided by the community advocate due to these limitations. Sixth, the results concerning HIV as a cause of death reported from informal verbal autopsies should be interpreted with caution. Lastly, in some cases records were missing, incomplete, or illegible due to the nature of record keeping in a public facility in a highly resource-constrained setting. In light of these limitations, there is a need to improve the capacity of public healthcare facilities to improve documentation and to ensure that children and youth have a right to identity including registration at birth, name and family relations as per the Convention on the Rights of the Child [9].

## Conclusion

In conclusion, most deaths among street-connected young people are preventable and require the urgent attention of service providers and policymakers to implement programs and services to prevent premature mortality. Particular attention should be given to designing HIV treatment programs for children and youth in street circumstances to improve their linkage and retention in care. With a large proportion of young people in street circumstances dying due to assault, child protection systems and specific policy to protect and uphold the rights of street-connected young people is urgently needed.

## Abbreviations

AMPATH: Academic Model Providing Access to Healthcare
CRC: Convention on the Rights of the Child
MTRH: Moi Teaching and Referral Hospital
UG: Uasin Gishu
UNOHCR: UN Office of the High Commissioner Human Rights
